# Association of zinc level and polymorphism in *MMP-7* gene with prostate cancer in Polish population

**DOI:** 10.1371/journal.pone.0201065

**Published:** 2018-07-23

**Authors:** Katarzyna Białkowska, Wojciech Marciniak, Magdalena Muszyńska, Piotr Baszuk, Satish Gupta, Katarzyna Jaworska-Bieniek, Grzegorz Sukiennicki, Katarzyna Durda, Tomasz Gromowski, Karolina Prajzendanc, Cezary Cybulski, Tomasz Huzarski, Jacek Gronwald, Tadeusz Dębniak, Rodney J. Scott, Jan Lubiński, Anna Jakubowska

**Affiliations:** 1 Department of Genetics and Pathology, International Hereditary Cancer Center, Pomeranian Medical University, Szczecin, Poland; 2 Read-Gene S.A., Grzepnica, Poland; 3 Strand Life Sciences, Bangalore, Karnataka, India; 4 School of Biomedical Sciences, University of Newcastle, Newcastle, Australia; 5 Division of Molecular Medicine, NSW Health Pathology, Newcastle, Australia; 6 Independent laboratory of Molecular Biology and Genetic Diagnostics, Pomeranian Medical University, Szczecin, Poland; University of South Alabama Mitchell Cancer Institute, UNITED STATES

## Abstract

**Introduction:**

Prostate cancer is one of the most commonly diagnosed malignancies among men in Western populations. Evidence reported in the literature suggests that zinc may be related to prostate cancer. In this study we evaluated the association of serum zinc levels and polymorphisms in genes encoding zinc-dependent proteins with prostate cancer in Poland.

**Methods:**

The study group consisted of 197 men affected with prostate cancer and 197 healthy men. Serum zinc levels were measured and 5 single nucleotide polymorphisms in *MMP-1*, *MMP-2*, *MMP-7*, *MMP-13*, *MT2A* genes were genotyped.

**Results:**

The mean serum zinc level was higher in prostate cancer patients than in healthy controls (898.9±12.01 μg/l vs. 856.6±13.05 μg/l, p<0.01). When compared in quartiles a significant association of higher zinc concentration with the incidence of prostate cancer was observed. The highest OR (OR = 4.41, 95%CI 2.07–9.37, p<0.01) was observed in 3^rd^ quartile (>853.0–973.9 μg/l). Among five analyzed genetic variants, rs11568818 in *MMP-7* appeared to be correlated with 2-fold increased prostate cancer risk (OR = 2.39, 95% CI = 1.19–4.82, p = 0.015).

**Conclusion:**

Our results suggest a significant correlation of higher serum zinc levels with the diagnosis of prostate cancer. The polymorphism rs11568818 in *MMP-7* gene was also associated with an increased prostate cancer risk in Poland.

## Introduction

Prostate cancer is one of the most commonly diagnosed malignancy among men in Western populations. In the last three decades the global incidence rates have been increasing, which can be partly explained by the introduction of screening tests such as PSA (prostate specific antigen) and surgical treatment for benign prostatic hyperplasia. PSA screening favors detection of cancers at early stages, mostly limited to organ, what is reflected in a slightly decreased mortality rate [1]. In 2015 in Poland more than 14,211 men were diagnosed with prostate cancer and 4,876 died. Most cases of prostate cancer are diagnosed in men older than 60. The 5-year survival rate which is 76.4% is quite high compared to other cancers [2]. In recent years many efforts have been made to identify potential risk factors for prostate cancer.

Thus far several genes have been associated with prostate cancer risk including *HOXB13* [3,4], *NBS1*[5,6] and *CHEK2* [7–10]. *HOXB13* missense mutation G84E accounts for 0.15% to 8% unselected prostate cancer cases and increases the risk up to 20-fold depending on the population [11] The frequency of *NBS1* mutations in sporadic prostate cancer cases is estimated to be between 0.15–2% depending on the population and is associated with 3-fold increase in disease risk [12, 8] The causative variant 1100delC in *CHEK2* is present in ~0.7% of unselected prostate cancers and is associated with 2-fold increase in risk [13].

Several other factors, such as hormones, smoking and diet, have been suggested to play a role in prostate cancer etiology [14]. One of the dietary factors that has been implicated in prostate cancer risk is zinc [15,16]. Zinc (Zn) is the essential micronutrient involved in many physiological processes that include enzyme activity, genomic stability, apoptosis, immunity, neurological function, response to oxidative stress, and cell signaling [17]. It has been observed that normal prostate cells accumulate high amounts of zinc while malignant cells are characterized by a dramatic (~85%) decrease in Zn levels [15]. Accumulated Zn inhibits the proliferation and growth of prostate cells. This micronutrient has also been shown to induce apoptosis and make malignant cells more sensitive to apoptosis [15]. Several *in vitro* studies revealed that zinc plays a protective role in carcinogenesis of prostate gland. Analyses performed on prostate tumour cell lines suggest that exposure to zinc and its intracellular accumulation inhibits human prostatic carcinoma cell growth, possibly due to induction of cell cycle arrest and mitochondrial apoptosis. The studies provided a strong evidence that the loss of capability to retain high levels of zinc is an important factor in the development and progression of malignant prostate cells [18,19]. However, other analyses on prostate cancer cell lines revealed that long-term treatment with zinc, in contrast to short-term, enhances chemoresistance, invasiveness and antioxidant capacity [20,21]. Epidemiological studies show mixed results on beneficial effect of zinc intake or supplementation. Some studies have revealed that use of Zn supplements or Zn rich diets were associated with reduced prostate cancer risk and mortality [22–24]. However, other studies suggest that long term Zn oversupply or high dietary intake positively correlates with increased prostate cancer risk [25,26].

Serum Zn level is an accurate biomarker of body Zn status as it well responses to dietary and supplemental intakes [27]. Most studies have suggested that serum Zn level in patients with prostate cancer is decreased comparing to those with benign hyperplasia or without a disease [28–33], whereas other demonstrated no associations [34]. Discrepancies between results may be caused by other factors that potentially influence Zn homeostasis. Some of these factors may be genetic alterations among proteins related to Zn, such as Zn metabolism proteins or metalloenzymes containing Zn ion in active site.

Metallothioneins (MTs) are proteins responsible for binding metals such as Zn and copper and then provide them to the needs of other proteins. They buffer cytosolic Zn, what is significant for maintaining redox status of the cells, as both decreased and increased Zn concentrations induce oxidative stress. High metallothionein expression has been observed in prostate cancer cells [35]. Matrix metalloproteinases (MMPs) are group of proteolytic enzymes containing Zn ions in their active sites, involved in extracellular matrix remodeling. MMPs are thought to play role in tumorigenesis and metastasis by regulating cell proliferation, apoptosis and angiogenesis. Expression level of MMPs genes in cells under physiological condition is low, however it is increased in malignant tissues [36]. Several functional single nucleotide polymorphisms (SNPs) located in promoter region, affecting gene transcription, have been identified among MMPs and MTs genes [37–42]. Reports suggested that these polymorphisms can be related to risk of various cancer [43–55].

In this study we sought to analyze to what extent variations in serum Zn level and polymorphisms in *MT2A*, *MMP-1*, *MMP-2*, *MMP-7* and *MMP-13* are related to prostate cancer among Polish men.

## Materials and methods

### Study participants

Prostate cancer group consisted of 197 consecutive patients diagnosed between 2012–2014 at the Clinical Hospital No 2. in Szczecin. Only patients without any prior history of malignancy and who were not treated before blood withdrawal were enrolled in this study. Controls were selected among men who were part of a population-based screening study performed by our center, in the West-Pomeranian region of Poland, to identify familial cancer syndromes. For each case one cancer-free control was selected. Controls were matched to cases with respect to year of birth (+/- 3 years), total number of prostate cancers among first degree relatives, number of other cancers among first degree relatives and smoking (status and number of pack/years). The characteristics of individuals enrolled in the study are shown in S1 Table. All participants gave informed written consent for the use of their samples and clinical data in research. The study was approved by Ethics Committee of the Pomeranian Medical University in Szczecin, Poland. Collected blood samples were fully anonymized prior to laboratory analyses.

### Samples preparation

From each participant two 10 ml samples of peripheral blood were obtained. The first sample was collected using the BD Vacutainer system (Becton Dickinson, USA) that utilised tubes containing a clot activator and dedicated to trace metals detection. Samples were incubated at room temperature for about 30 min. to obtain a clot and then centrifuged at 1300 x g for 12 minutes. Serum was then transferred to cryovials and stored at -80°C until analysis. Before Zn measurement the serum sample was thawed, mixed and centrifuged at 5000 x g for 5 minutes.

A second sample of peripheral blood was taken for DNA isolation using the BD Vacutainer system with tubes containing K2-EDTA. The DNA isolation was performed using the detergent method as previously described [56]. After isolation the DNA samples were stored at 4°C prior to analysis.

### Measurement of Zn level

Zn concentration in serum samples was measured using the inductively coupled plasma mass spectroscopy (ICP-MS) technique with a NexION 350D (PerkinElmer, USA) instrument. Before each analytical run the instrument was standardized to the manufacturers’ specifications. If the performance of the spectrometer did not meet the appropriate specification, the instrument was re-calibrated. The blank reagent was used to dilute standards and samples, and consisted of 0,65% HNO_3_ Suprapur Grade (Merck, Germany) and 0,002% Triton X-100 (PerkinElmer, USA). Germanium (Ge74) was set as an internal standard. Calibration curve standards (1, 2, 5 and 10 μg/l) were prepared by diluting 10 mg/l Multi-element Calibration Standard 3 (PerkinElmer Pure Plus, USA) with the blank reagent. An external calibration method was used and a correlation coefficient for Zn detection (always greater than 0.999) was used to determine the final serum Zn concentration. Before the analysis serum samples were thawed and centrifuged (1500 x g, 15 minutes) and the supernatant was diluted 100 fold with blank reagent and directly measured. ClinChek^®^ Serum Control Level I (Recipe, Germany) was used as a reference standard. The reference material was measured after every sixth sample to ensure against measurement drift. If the drift in reference material measurements was greater than 5% the measurements of all 6 samples were repeated after correction.

### Molecular analysis

Five SNPs in five genes coding Zn-related proteins were analyzed: rs1799750 in *MMP-1*, rs243865 in *MMP-2*, rs11568818 in *MMP-7*, rs2252070 in *MMP-13* and rs28366003 in *MT2A*. Genotyping was performed by Real-Time PCR using Taqman probes. The rs243865 in *MMP-2*, rs11568818 in *MMP-7*, rs2252070 in *MMP-13* and rs28366003 in *MT2A* were analyzed using pre-designed Genotyping Assays. For analysis of rs1799750 in *MMP-1* a customized assay was used.

The reaction mix for analysis of each sample consisted of GoTaq^®^ Probe qPCR Master Mix (Promega, USA), TaqMan Genotyping Assay x 40 (Applied Biosystems, USA) and deionized water in accordance to TaqMan Genotyping Assay’s manufacturer’s instructions. The reaction mix and sample DNA were placed in 384 well plates (Axygen, USA) and Real-Time PCR reactions were performed on a LightCycler^®^ 480 (Real-Time PCR System, Roche Diagnostics, USA). For genotyping data analysis LightCycler480 Basic SoftwareVersion1.5 was used (Roche Diagnostics, USA).

### Statistical analysis

Comparison of year of birth, pack-years, number of prostate cancers among I° relatives and Zn concentration between cases and controls was performed using Mann-Whitney U-test.

To evaluate an association of serum Zn levels with prostate cancer incidence, the individuals were assigned to one of four quartiles based on the distribution of Zn levels in the entirestudy group (cases and controls). Quartile I, with lowest Zn concentrations, was considered as a ‘reference’ to which the cancer rates in other quartiles were compared. Evaluation of the association of tested polymorphisms with cancer risk was performed by comparing the frequencies of genotypes among patients and healthy subjects. The most frequent homozygous allele was considered as the ‘reference’ genotype.

The odds ratios (ORs) and corresponding 95% confidence intervals (95% CI) for Zn levels were estimated by multivariable logistic regression and adjusted for 5 tested SNPs. The odds ratios (ORs) and corresponding 95% confidence intervals (95% CI) for each SNP were also estimated by multivariate logistic regression model and adjusted for Zn levels. Analyses were performed using R Project for Statistical Computing (ver. 3.3.3.).

## Results

The mean Zn level was significantly higher in the group of prostate cancer patients compared to the cancer-free controls (898.9±12.01 μg/l vs 856.6±13.05 μg/l, p<0.01). Analysis in quartiles demonstrated that Zn levels were associated with prostate cancer incidence. Odds ratios in 3 quartiles (>753.9–853.0, >853.0–973.9 and >973.9 μg/l) were significantly higher compared to the lowest quartile (<753.9 μg/l) (S2 Table, S1 Fig). The highest OR (OR = 4.41, 95%CI 2.07–9.37, p<0.01) was observed in 3^rd^ quartile (>853.0–973.9 μg/l).

Among the five analyzed genetic variations one polymorphism (rs11568818) in *MMP-7* appeared to be correlated with prostate cancer risk. The GG genotype was associated with 2-fold increased cancer risk compared to AA genotype (OR = 2.03, 95% CI 1.07–3.86, p = 0.03) (S3 Table). For all other polymorphisms assessed in this study, no association with cancer risk was observed.

## Discussion

In our retrospective case-control study we sought to investigate whether serum Zn levels could be a useful marker for prostate cancer risk in Polish men. We also determined if polymorphisms in several Zn-dependent genes (*MT2A*, *MMP-1*, *MMP-2*, *MMP-7*, *MMP-13*) contributed to prostate cancer risk. We found that serum Zn levels were significantly higher in prostate cancer patients than in controls (p = 0.01). Moreover it was shown that higher Zn levels are associated with a greater prostate cancer incidence (S2 Table). One of the analyzed polymorphisms, rs11568818 in *MMP-7*, was also associated with increased prostate cancer risk (p = 0.03).

Several studies correlating serum Zn concentration and prostate cancer have been conducted [28–34]. Analyses performed in India, China, Turkey and Poland suggested an association between a lower serum Zn levels and a decrease in prostate cancer risk [28,30–33]. Two separate studies in Indian population including 10 cases/20 controls and 18 cases/20 controls revealed that serum Zn levels in prostate cancer patients is lower compared to controls (596 μg/l vs. 945 μg/l, p<0.01 and 630 μg/l vs. 865 μg/l, p<0.05) [32,28]. A similar association was found in Turkish prostate cancer patients where 30 cases and 32 controls (Zn level 0.713 in patients vs. 2.945 in controls, respectively, p = 0,001) [33]. Chinese analyzes conducted in a group of men with PSA levels between 4–10 ng/ml from 42 cases and 101 controls revealed Zn levels were decreased in cancer patients compared to controls (83 ng/ml vs. 97.8 ng/ml, respectively, p = 0.001) [31]. Moreover, they found increased probability of detecting prostate cancer at lower Zn concentrations (40 ng/ml–600 ng/ml vs. >1000 ng/ml: OR = 5, 95%CI 5.44–56.69). In a Polish study of 94 cancer cases and 80 controls with benign prostate hyperplasia, a significantly lower serum Zn level was observed in prostate cancer patients compared to controls, but only in the subgroup of men at ages ≤65 years (700 ng/ml vs. 980 ng/ml) and not in those >65 years (690 ng/ml vs. 840 ng/ml) [30]. Despite generally consistent results, all these studies suffered from the small sample sizes and differed in terms of patient selection. In the current study the mean serum Zn concentration was significantly higher in the 197 prostate cancer patients compared to the 197 matched healthy controls (898.9 μg/l vs. 856.6 μg/l, p <0.01). Moreover, Zn concentrations >753.9 μg/l were associated with higher ORs of prostate cancer. Compared to Zn concentrations <753.9 μg/l (1^st^quartile), the probability of prostate cancer diagnosis was elevated 2-fold (OR = 1.96, p = 0.04) for a concentration of 753.90 μg/l–853.00 μg/l (2^nd^ quartile), more than 3-fold (OR = 3.39, p <0.01) for a concentration >973.90 μg/l (4^th^ quartile) and more than 4-fold (OR = 4.41, p <0.01) for a concentration 853.00 μg/l–973.90 μg/l (3^rd^ quartile) (S2 Table). Discrepancies between the results of our study and previous analyzes may be due to several factors, that include differences in sample size, selection of patients enrolled in the study, or sample collection and analysis. In five analyzes the study groups were small and varied between 10 to 94 prostate cancer patients, and 20 to 101 controls [28,30–33]. In three studies it is not clear which criteria were used to enroll patients in the study [28,32,33]. The Chinese analysis [31] only included men selected on elevated PSA (4–10 ng/ml). Whereas, in the Polish study, statistically significant association of zinc concentration with prostate cancer was observed only the group of men ≤65 years of age, additionally, the control group were all diagnosed with benign prostatic hyperplasia. [30]. The discrepancies between studies could be also explained by population-related differences, which have also been observed in a large prospective study [34]. In an analysis of 392 prostate cancer patients and 783 healthy controls identified from 215,000 adults including five ethnic/racial groups (African Americans, Native Hawaiians, Japanese Americans, Latinos, and whites), a suggestion of a possible ethnic-specific association between high Zn levels and cancer risk was reported [34]. A positive association was found in Japanese Americans (OR for the highest vs. the lowest tertile = 2.59, 95% CI 1.09–6.17) and Latinos (OR = 2.74, 95% CI 1.05–7.10), whereas no association was observed in African Americans and white caucasians [34]. The observed heterogeneity may be explained by genetic diversity in genes encoding Zn-dependent proteins. Zinc has been previously shown to correlate with prostate carcinogenesis, but *in vitro* studies on cell lines show mixed results on its either protective or harmful effect [18,19,20,21]. Results of our analysis corresponds with recent studies, where long-term zinc exposure was associated with enhanced malignancy in prostate cancer cells [20,21]. Reports by Holubova et al. [20] and Kratochvilova et al. [21] showed that long-term zinc administration to 22Rv1 and PC-3 prostate carcinoma cell lines enhances expression of different genes than short-term administration, which leads to chemoresistance to zinc and cisplatin, increased oxidative stress tolerance and make malignant cells more invasive. In our study in addition to zinc concentration we analyzed functional polymorphisms localized in metallothionein *MT2A* and four metalloproteinases: *MMP-1*, *MMP-2*, *MMP-7*, *MMP-13*. We found significant association of rs11568818 in *MMP-7* gene with higher prostate cancer risk.

The rs11568818 in *MMP-7* (-181 A> G) is located in the promoter region and has been shown to influence gene expression [39,57]. The results of our study revealed a significant association of the GG genotype with a 2-fold higher risk of prostate cancer (OR = 2.03, p = 0.03) (S3 Table). This polymorphism was analyzed previously in two other studies, both of which reported inconclusive results [58,59]. Eeles et al. (2013) in a multicenter analysis of about 40,000 individuals European ancestry from 32 studies showed an inverse association of rs11568818 in *MMP-7* with prostate cancer risk (OR = 0.91, p = 2.4x10^-10^) [58]. In contrast, Hoffmann et al. (2017) in a multicenter analysis of about 34,000 non-Hispanic white individuals reported an association of rs11568818 with a higher prostate cancer risk (OR adjusted for PSA level = 1.12, p = 4.9x10^-6^) [59]. Both studies were multicenter and similarly sized, however, they differed in the type and structure of the analyzed populations. A meta-analysis of 24 studies revealed the association of rs11568818 in *MMP-7* with cancer risk differed among ethnicities [60]. Our observation, that GG genotype in rs11568818 is associated with increased prostate cancer risk corresponds with results from the earlier functional analyses. The *MMP-7* gene has been already suggested to correlate with prostate carcinogenesis. *In vitro* study on prostate cancer cells showed that *MMP-7* gene transcription is significantly upregulated in human prostate tumours comparing to normal tissue [61]. Moreover, analyses in rat models of prostate cancer revealed that *MMP-7* is upregulated during carcinogenesis [62,63]. The increased transcription of *MMP-7* was also associated with development and progression of prostate cancer in human both *in vitro* [64,65] and *in vivo* [64]. Analyses performed in cell lines revealed that G allele in rs11568818 correlates with higher MMP-7 expression and enzyme activity [39,66]. Our result supports the observations about the role of MMP-7 in prostate cancer development.

In our analysis no association between other tested polymorphisms (rs1799750 in *MMP-1*, rs243865 in *MMP-2*, rs2252070 in *MMP-13* and rs28366003 in *MT2A)* and prostate cancer risk was shown. There are limited available data about the above variations and results are inconsistent. The rs243865 in *MMP-2* was previously reported to correlate with increased prostate cancer risk among Indian population [47], however study conducted in Iran [67] did not confirm this finding. The only study on rs28366003 in *MT2A* was conducted in Poland [68] and showed that this polymorphism may contribute to high risk of prostate cancer. Our findings did not confirm the association between these two polymorphism with prostate cancer risk in Polish population.

The main advantage of our study is the complex analysis of serum Zn levels and polymorphisms in genes encoding Zn-dependent proteins in a well-annotated group of prostate cancer patients and controls. Cases and controls were matched in respect to significant factors that possibly modify prostate cancer risk such as age (birth year) or family history (number of prostate and other cancers in first degree relatives) in order to avoid influence of these factors on the results presented. We cannot exclude, however, other environmental or genetic variables that were beyond the scope of this study. Furthermore, material from cancer patients was collected at the time of diagnosis, before any treatment or surgery was undertaken in order to limit the possible influence of medical intervention on serum Zn levels.

The limitation of our study is the retrospective case-control design. Such an approach does not allow for the exclusion of malignancy on serum Zn levels or to assess whether higher serum Zn levels promoted prostate cancer or was a consequence of it. However our results indicate that high serum Zn levels may be a marker that can be used to identify patients at higher risk of disease, to who further testing should be offered to confirm or exclude prostate cancer. Moreover, genotyping of rs11568818 in *MMP-7* could identify those patients who have an increased risk of developing prostate cancer in the Polish population.

## Conclusion

Our results suggest a significant correlation between serum Zn levels and the risk of prostate cancer. Analyses of polymorphisms in genes encoding Zn-associated proteins revealed a significant correlation of rs11568818 in *MMP-7* gene with an increased prostate cancer risk. However, a large prospective study on Zn levels and genetic variations in Zn-dependent proteins is necessary to determine their precise role in cancer pathogenesis.

## Supporting information

S1 Table Characteristic of prostate cancer cases and controls included in the study. (PDF)

S2 Table Serum Zn concentration and prostate cancer occurrence. (PDF)

S3 Table Polymorphisms and prostate cancer risk. (PDF)

S1 Fig The odds ratios for prostate cancer incidence depending on serum Zn level. (TIF)
