# Supplementary material for: Association of zinc level and polymorphism in MMP-7 gene with prostate cancer in Polish population
Source: PLoS One. 2018 Jul 23;13(7):e0201065. doi: 10.1371/journal.pone.0201065 (PMC6056054; doi:10.1371/journal.pone.0201065)
Supplement: S1 Table — (PDF) [file pone.0201065.s001.pdf]

S1 Table. Characteristic of prostate cancer cases and controls included in the study.

**Characteristic**	**Cases (n=197)**	**Controls (n=197)**	**p-value***
Mean year of birth (range)	1946 (1926 – 1962)	1946 (1931-1960)	0.60
Smoking, n (%)			
Yes (current + past)	136 (69)	132 (67)**	-
No (never smokers)	61 (31)	65 (33)**	-
Pack-years (range)	15.73 (0-80)	15.39 (0-80)	0.94
Prostate cancer among I° relatives, n (%)			
Yes	20 (10)	20 (10)	-
No	177 (90)	177 (90)	-
