# Supplementary material for: Association of zinc level and polymorphism in MMP-7 gene with prostate cancer in Polish population
Source: PLoS One. 2018 Jul 23;13(7):e0201065. doi: 10.1371/journal.pone.0201065 (PMC6056054; doi:10.1371/journal.pone.0201065)
Supplement: S2 Table — (PDF) [file pone.0201065.s002.pdf]

S2 Table. Serum Zn concentration and prostate cancer occurrence.

**Zn level (µg/l)**	**Cases (%) n=197**	**Controls (%) n=197**	**OR* (95% CI)**	**p-value**
<753.9	35 (18)	63 (32)	1	-
753.9-853.0	46 (23)	52 (27)	**1.96 (1.02-3.76)**	**0.04**
853.0-973.9	61 (31)	38 (19)	**4.41 (2.07-9.37)**	**<0.01**
>973.9	55 (28)	44 (22)	**3.39 (1.63-7.26)**	**<0.01**

*Multivariable logistic regression adjusted for 5 tested SNPs.
