# Supplementary material for: Association of zinc level and polymorphism in MMP-7 gene with prostate cancer in Polish population
Source: PLoS One. 2018 Jul 23;13(7):e0201065. doi: 10.1371/journal.pone.0201065 (PMC6056054; doi:10.1371/journal.pone.0201065)
Supplement: S3 Table — (PDF) [file pone.0201065.s003.pdf]

S3 Table. Polymorphisms and prostate cancer risk.

	**Cases (%), n=197**	**Controls (%), n=197**	**OR* (95%CI)**	**p-value**
***MMP-1* rs1799750**				
1G/1G	56 (28.4)	54 (27.4)	1	-
1G/2G	105 (53.3)	90 (45.7)	1.24 (0.76-2.02)	0.39
2G/2G	36 (18.3)	53 (26.9)	0.73 (0.41-1.28)	0.27
***MMP-2* rs243865**				
CC	104 (52.8)	101 (51.3)	1	-
CT	79 (40.1)	78 (39.6)	0.95 (0.61-1.48)	0.82
TT	14 (7.1)	18 (9.1)	0.76 (0.32-1.82)	0.54
***MMP-7* rs11568818**				
AA	59 (29.9)	76 (38.6)	1	-
AG	100 (50.8)	97 (49.2)	1.27 (0.80-2.02)	0.31
GG	38 (19.3)	24 (12.2)	**2.03 (1.07-3.86)**	**0.03**
***MMP-13* rs2252070**				
TT	92 (46.7)	104 (52.8)	1	-
CT	87 (44.2)	78 (39.6)	1.16 (0.74-1.81)	0.51
CC	18 (9.1)	15 (7.6)	1.39 (0.64-3.02)	0.40
***MT2A* rs28366003**				
AA	179 (90.9)	176 (89.3)	1	-
AG + GG**	18 (9.1)	21 (10.7)	0.73 (0.35-1.52)	0.40

*Multivariable logistic regression adjusted for Zn level

** Genotypes were summarized because of low number of GG genotype
