# Supplementary figures and images for: Association of zinc level and polymorphism in MMP-7 gene with prostate cancer in Polish population
Source: PLoS One. 2018 Jul 23;13(7):e0201065. doi: 10.1371/journal.pone.0201065 (PMC6056054; doi:10.1371/journal.pone.0201065)

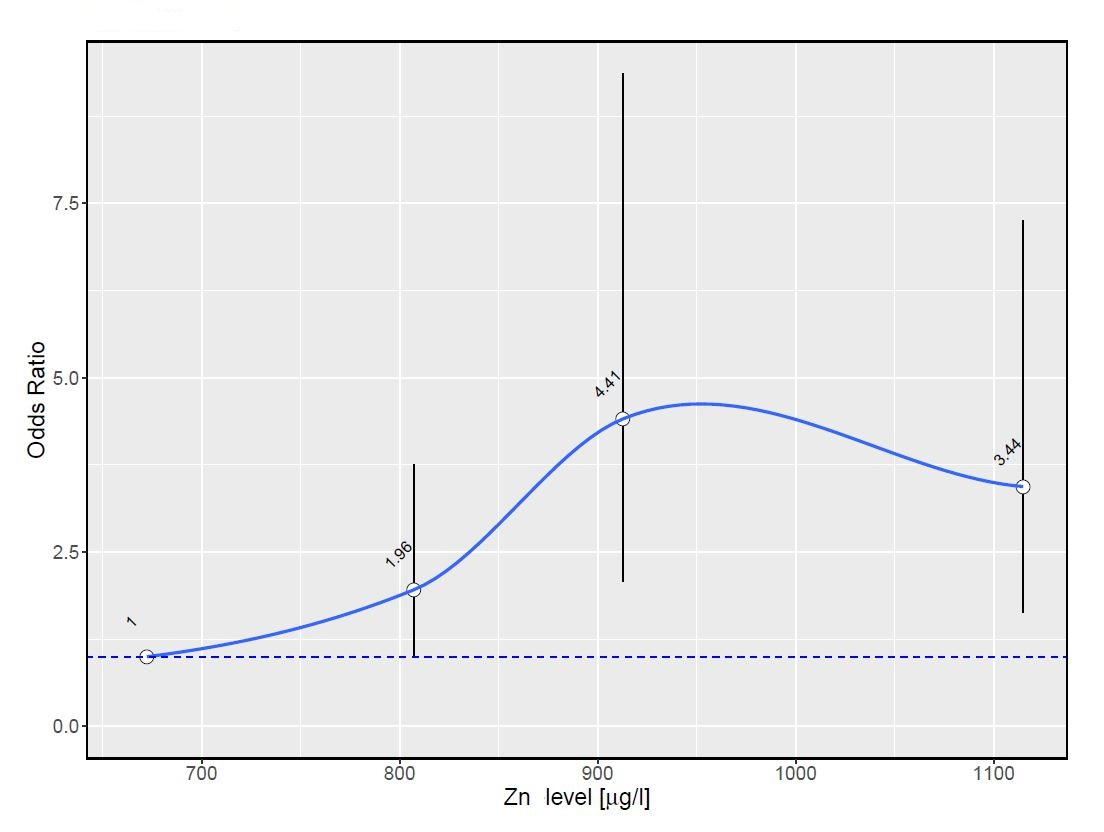

Supplement: S1 Fig — (TIF) [file pone.0201065.s004.tif]
